# Photochemical tissue bonding with chitosan adhesive films

**DOI:** 10.1186/1475-925X-9-47

**Published:** 2010-09-08

**Authors:** Antonio Lauto, Damia Mawad, Matthew Barton, Abhishek Gupta, Sabine C Piller, James Hook

**Affiliations:** 1 Nanoscale-Organization and Dynamics Group, School of Biomedical and Health Sciences, School of Medicine, University of Western Sydney, NSW 1797, Australia; 2 ARC Centre of Excellence for Electromaterials Science, Intelligent Polymer Research Institute, University of Wollongong, NSW, Australia; 3 School of Chemistry, University of New South Wales, Sydney, NSW, 2052, Australia

## Abstract

**Background:**

Photochemical tissue bonding (PTB) is a promising sutureless technique for tissue repair. PTB is often achieved by applying a solution of rose bengal (RB) between two tissue edges, which are irradiated by a green laser to crosslink collagen fibers with minimal heat production. In this study, RB has been incorporated in chitosan films to create a novel tissue adhesive that is laser-activated.

**Methods:**

Adhesive films, based on chitosan and containing ~0.1 wt% RB were manufactured and bonded to calf intestine by a solid state laser (λ = 532 nm, Fluence~110 J/cm^2^, spot size~0.5 cm). A single-column tensiometer, interfaced with a personal computer, tested the bonding strength. K-type thermocouples recorded the temperature (T) at the adhesive-tissue interface during laser irradiation. Human fibroblasts were also seeded on the adhesive and cultured for 48 hours to assess cell growth.

**Results:**

The RB-chitosan adhesive bonded firmly to the intestine with adhesion strength of 15 ± 2 kPa, (n = 31). The adhesion strength dropped to 0.5 ± 0.1 (n = 8) kPa when the laser was not applied to the adhesive. The average temperature of the adhesive increased from 26°C to 32°C during laser exposure. Fibroblasts grew confluent on the adhesive without morphological changes.

**Conclusion:**

A new biocompatible chitosan adhesive has been developed that bonds photochemically to tissue with minimal temperature increase.

## Background

Suturing is the standard procedure for closing wounds in surgical operations. Significant complications can however occur postoperatively such as inflammation and scar tissue formation, often due to non-absorbable sutures [1]. Manual dexterity is also needed when suturing in keyhole operations that are time consuming. Alternative methods for wound closure have been developed and refined in the past decades. Laser tissue welding (LTW), for example, is a technique that provides tissue sealing using laser energy. The laser beam penetrates into the interface of two tissue edges, previously overlapped, and crosslinks the collagen fibers sealing the wound [2]. A variety of lasers have been used to repair blood vessels, peripheral nerves, intestine and the cornea in experimental and clinical trials [3-6]. The wavelengths employed for LTW are usually in the mid-infrared region as water in tissues can absorb the laser and produce heat, which fuses and bonds collagen at 60-65°C [2]. The laser can also be selectively absorbed by a dye applied between two tissue stumps. Indocyanine green and rose bengal are among the biocompatible dyes that are currently used by researchers [6,7]. However, there is a fundamental difference between these two dyes; the formal absorbs the laser at 810 nm in an exothermic reaction while rose bengal photochemically cross-links collagen, without significant heat production (λ = 532 nm) [8]. The major and crucial advantage of photochemical tissue bonding (PTB) is thus the lack of significant increase in temperature inside the tissue, which avoids thermal damage. The bonding strength of tissue that is repaired with laser and RB is comparable to the strength of photo-thermal LTW; nevertheless tissue can suffer thermal injury in the latter case as temperature rises to 60-70°C [9,10]. PTB and LTW are therefore two distinct methods of wound closure. Other sutureless techniques for tissue repair include laser-activated glues (usually in a liquid or gel forms) and adhesive films. Albumin based glues, also known as solders, and chitosan adhesive films have been developed and applied to repair a range of tissues, including dura mater, peripheral nerves, bowels, blood vessels and urethra [11-15]. Solders and adhesives are usually placed across two tissue edges and laser irradiated to seal the wound. The laser (λ = 810 nm) is absorbed by IG, which is the typical dye incorporated in these biomaterials. The produced heat is essential to bond tissue to solders or chitosan adhesives. Unfortunately, the tissue damage associated with the exothermal absorption of the dye can be detrimental because tissue temperature often exceeds 70°C [16]. At this temperature, albumin and collagen molecules denature and crosslink together. The collateral thermal damage inflicted on tissue is currently a major obstacle for the implementation of these sutureless techniques. In the present study, we describe for the first time a novel chitosan adhesive film that comprises RB as the laser-absorbing dye. The film is biocompatible and successfully bonded *in vitro *to calf small intestine with a modest increase of temperature (~6°C).

## Methods

### Adhesive Film Preparation

All chemicals were purchased from Sigma-Aldrich (Sigma, St. Louis, MO, USA) and used without any further purification. Deacetylated chitosan (≥ 85%) from crab shells was dissolved at a concentration of 1.7% w/v in deionised water (50 mL) containing acetic acid (2% v/v) and Rose Bengal (RB, 0.014% w/v). RB was added to the chitosan solution in order to explore a possible non-thermal photochemical bonding between the tissue and chitosan adhesive [7,8]. The viscous chitosan solution was stirred for 2 weeks at room temperature (~25°C) in a vial shielded from light to avoid photo-bleaching of RB. RB was not readily soluble in the acidic solution and thus required prolonged stirring. The homogenized chitosan solution was then spread evenly (thickness ~1 mm, surface area ~12 cm^2^) over a sterile and dry perspex plate and allowed to dry for 2 weeks at room temperature under clean conditions and atmospheric pressure. The resulting chitosan film, which was bright rose in color, was carefully detached from the plate without damage. A digital micrometer measured the film thickness that ranged from 15 to 20 μm. All films were thereafter cut in rectangular strips (~10 × 6 mm), placed between sterile glass slides to preserve their flat shape and wrapped in aluminum foil for light shielding at room temperature. Hereafter, the RB-chitosan film will be referred to as the "rose adhesive".

### Adhesive Optical Attenuation

A UV-Visible spectrophotometer was used to measure the laser attenuation at 532 nm within the rose adhesive film and RB solution in deionised water. The wavelength of 532 nm is strongly absorbed by RB in phosphate buffer solution (PBS) and corresponds to the laser wavelength used for tissue repair [17]. A rose adhesive was fixed inside a quartz cuvette and its attenuated spectrum was recorded over the range of 400-800 nm. Spectra of chitosan films without RB were also recorded to serve as control samples. The attenuation length of the adhesive (~63% intensity attenuation) was calculated by assuming the validity of Beer's law: I = I_0_e**^-*Ax*^**, where I_0 _is the incident beam intensity, $\frac{1}{A}$ is the attenuation length and *x *is the film thickness.

### The Laser System

The adhesion of the rose adhesive was activated by a diode-pumped solid state laser that was coupled to a multimode optical fiber (CNI Lasers, China). The fiber was inserted in a hand-held probe to provide easy and precise beam delivery by the operator. The laser emitted a power of 180 mW at 532 nm in continuous wave, with a fiber core diameter of 200 μm and numerical aperture of 0.22. A Teflon "spacer" was mounted on the fiber probe to ensure the surgeon irradiated tissue from the same distance with a beam spot size of ~ 0.5 cm. Because the laser is not eye safe (Class IV), safety goggles were worn during the operations.

### In Vitro PTB

**Figure 1:**
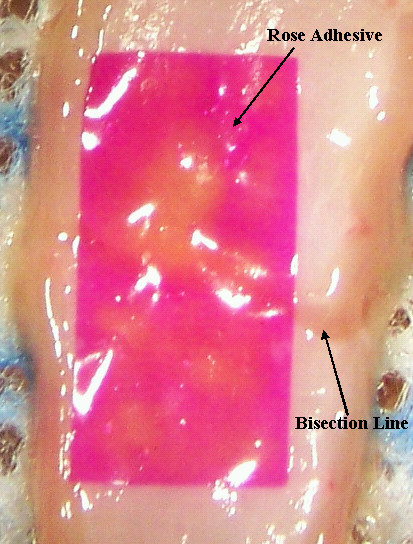
**The rose adhesive is bonded to calf intestine after laser exposure**. In this image, the laser irradiated deliberately selected spots to show up the RB photo-bleaching. Uniform irradiation was however applied on the adhesive during the tissue-bonding study.

The adhesive strength of the bandage was tested *in vitro *on calf intestine, which was harvested immediately after animal euthanasia and stored at -80°C. Prior to use, tissue was immersed in deionized water for 15 minutes to defrost and hydrate at room temperature. Intestine sections (~2 × 1 cm) were bisected by a full thickness incision with a #10 blade under an operating microscope (X 20). The intestine was kept moist using deionized water; excess water was absorbed with cotton tips prior to tissue repair. The incision stumps were approximated end -to -end and a ~10 × 6 mm rose adhesive was positioned across the incision on the serosa layer with microforceps ensuring full contact with the intestine (Figure 1). Thereupon, the operator spot-irradiated the adhesive ensuring each spot was irradiated for ~ 5 seconds before moving the beam to the adjacent spot. This procedure guaranteed that the laser beam scanned the whole surface area of the adhesive several times (Table 1). The laser was absorbed by the RB dye that discolored in the adhesive during PTB (Figure 1). The laser fluence (~110 J/cm^2^) was similar to the ones (70-134 J/cm^2^) used in a previous *in vivo *study. In that instance, the anastomosis of rat arteries was accomplished with no thermal damage by performing PTB with a rose bengal solution [7].

**Table 1:** Laser parameters for PTB

	N	Area (mm^2^)	Power (W)	Time (s)	Fluence (J/cm^2^)	I (W/cm^2^)	Max Load/Area (kPa)
**Adhesive+Laser**	31	60 ± 10	0.18 ± 0.03	365 ± 5	110	~0.9	15.1 ± 1.2

**Adhesive**	8	60 ± 10	NA	NA	NA	NA	0.5 ± 0.1

*N*, sample number; *Area*, surface area of the rose adhesive (mean ± maximum error); *Power*, laser power (mean ± maximum error); *Time*, irradiation time (mean ± maximum error), *Fluence*, average laser fluence, *I*, estimated irradiance; *Max Load/Area*, maximum load at failure of the repaired tissue divided by the adhesive surface area, (mean ± SE).

### Tensile Test

**Figure 2:**
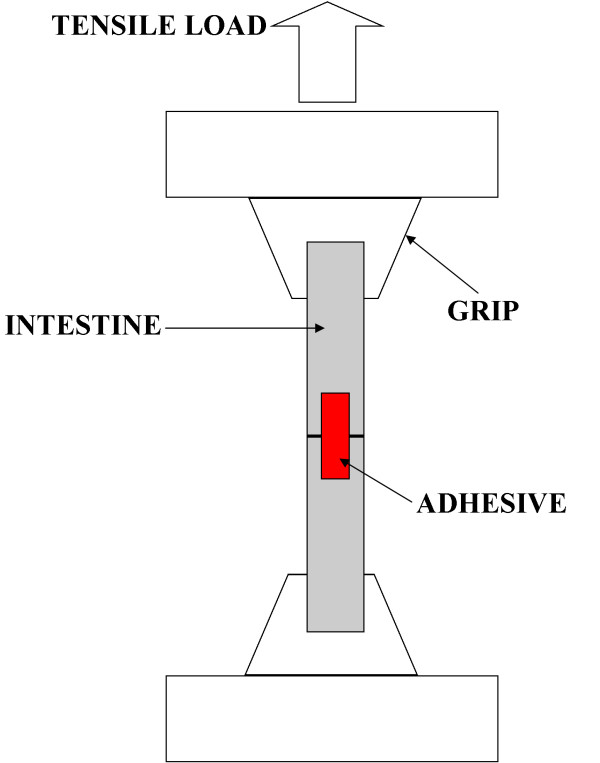
**Schematic of the tensile test used to estimate the bonding strength of the rose adhesive**.

To assess the tissue bonding strength, each intestine section (sample) was tested after PTB repair with a calibrated single-column tensiometer (Instron, MA, USA), which was interfaced with a personal computer. Tissue was maintained in wet gauze before the tensile test to mimic *in vivo *conditions and avoid sample desiccation. A sample was clamped to the tensiometer using mechanical grips, which moved at a rate of 22 mm/min until the two tissue stumps separated (Figure 2). The maximum load at which the stumps separated (80% load drop) was recorded with Merlin IX software. Strips of rose adhesive were also applied to bisected tissue, as described in the previous paragraph, but without laser irradiation to serve as a control group. Data were analyzed with the unpaired two tails Student's t-test.

### Temperature Measures

The temperature increase underneath the rose adhesive, due to the laser beam, was measured in a separate experiment with an insulated K-type thermocouple (diameter = 0.25 mm, response time = 0.1 s). This was positioned between the intestine and the rose adhesive, as described in a previous study [18]. The thermocouple was inserted through a hole punched from the bottom part of the intestine with a 10 gauge needle. The operator ensured, through an operative microscope, that there was full contact between the adhesive and the thermocouple. The thermocouple was calibrated and connected to a digital multimeter to record and store temperature data every 5 sec. The adhesive was irradiated at a power level of 180 ± 5 mW and data were recorded for 30 s while the beam was directed in the thermocouple proximity (~1 mm offset). A sudden rise of temperature was noticed whenever the laser illuminated the thermocouple. For this reason the laser spot size on the adhesive was reduced to ~2 mm and particular care was taken to avoid direct irradiation of the thermocouple. After irradiation, the adhesive was pulled with microforceps to ensure that tissue adhesion had occurred.

### Adhesive Cytotoxicity

Cells were grown on the rose adhesive to qualitatively assess its cytotoxicity. Human fibroblast from neonatal foreskin (HFNF) were cultured and maintained at 37°C in a humidified atmosphere containing 5% CO_2_. Cells were grown in 2 mL DMEM with 10% FCS, 100 units/mL penicillin-streptomycin and 2% L-glutamate. HFNF were seeded at 10 × 10^5 ^cells/well in a 6-well culture plate and grown for 48 h. A strip of rose adhesives was sterilized with 100% ethanol, washed in PBS and placed in the well before adding the cells. Control wells consisted of cells in medium without the adhesive. The experiment was done in triplicate.

## Results

### Adhesive Optical Attenuation

**Figure 3:**
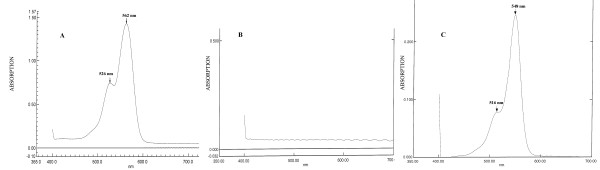
**(A) The absorption spectrum of the rose adhesive shows two peaks at 526 and 562 nm**. The green laser (λ = 532 nm) was thus strongly absorbed by the adhesive during PTB. (B) The absorption spectrum of the chitosan film without RB. These films poorly attenuated visible light. (C) The absorption spectrum of RB dissolved in deionised water ([RB]~5*10^-6 ^molar). The peaks are shifted to 516 and 548 nm.

The rose adhesive absorbed strongly the laser at 532 nm and the corresponding attenuation length was 12.4 ± 2.0 μm (n = 5, Figure 3A). In contrast, chitosan films without RB attenuated weakly the laser ($\frac{1}{A}$ = 162.8 ± 21.7 μm, n = 3), likely due to scattering (Figure 3B). Assuming minor scattering and reflection, we may ascribe to RB the efficient absorption of the laser energy at 532 nm inside the adhesive. The absorption peaks of the rose bengal in water solution (n = 3) occurred at λ_1_= 548 nm and λ_2_= 516 nm; while these peaks were respectively shifted to λ_1_= 562 nm and λ_2_= 526 nm in the adhesive films. It appears from the spectra plots that no significant aggregation of RB has occurred in the films (Figures 2A and 2C).

### In Vitro PBT and Tensile Tests

The rose adhesive bonded firmly to the intestine upon laser irradiation achieving a maximum load at failure of 0.91 ± 0.07 N (mean ± SE, n = 31). The adhesive separated from tissue without cohesive breaks in all tests. For this reason, the adhesive strength was estimated as the maximum load divided by the adhesive surface area, namely, 15.1 ± 1.2 kPa (mean ± SE, n = 31). The non-irradiated rose adhesive bonded much less to tissue (0.5 ± 0.1 kPa, n = 8) and other seven non-irradiated samples could not be tested due to the weak adhesion strength. There was a very significant statistical difference between the adhesion strength of these two groups (p < 10^6^).

### Temperature Measures

**Figure 4:**
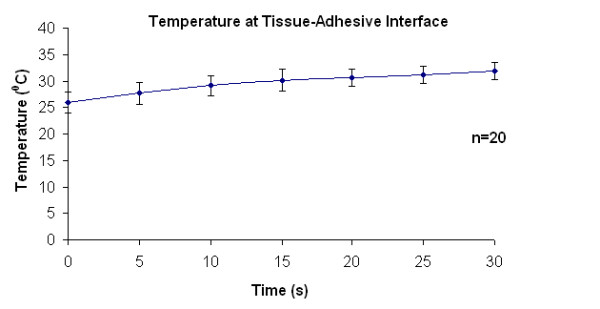
**Temperature profile of the rose adhesive at the tissue interface during PTB**. The adhesive temperature increased modestly from 26 to 32°C (n= 20, mean ± SD).

The profile of the recorded temperatures is plotted in Figure 4. The estimated average temperature of the rose adhesive remained below 32°C during laser irradiation (n = 20). The temperature increased by ~6°C during the 30 seconds of laser activation. These results showed the interaction of the laser with adhesive RB is mostly non-exothermal, in agreement with previous reports [7,19,20]. The thermal mass of the thermocouple should not affect significantly the measured temperatures at the tissue/adhesive interface, due to its small diameter (0.25 mm). Despite avoiding direct irradiation of the thermocouple, some light was scattered towards the thermocouple by the adhesive and this may have contributed to the temperature increase. It is problematic to estimate such contribution.

### Adhesive Cytotoxicity

**Figure 5:**
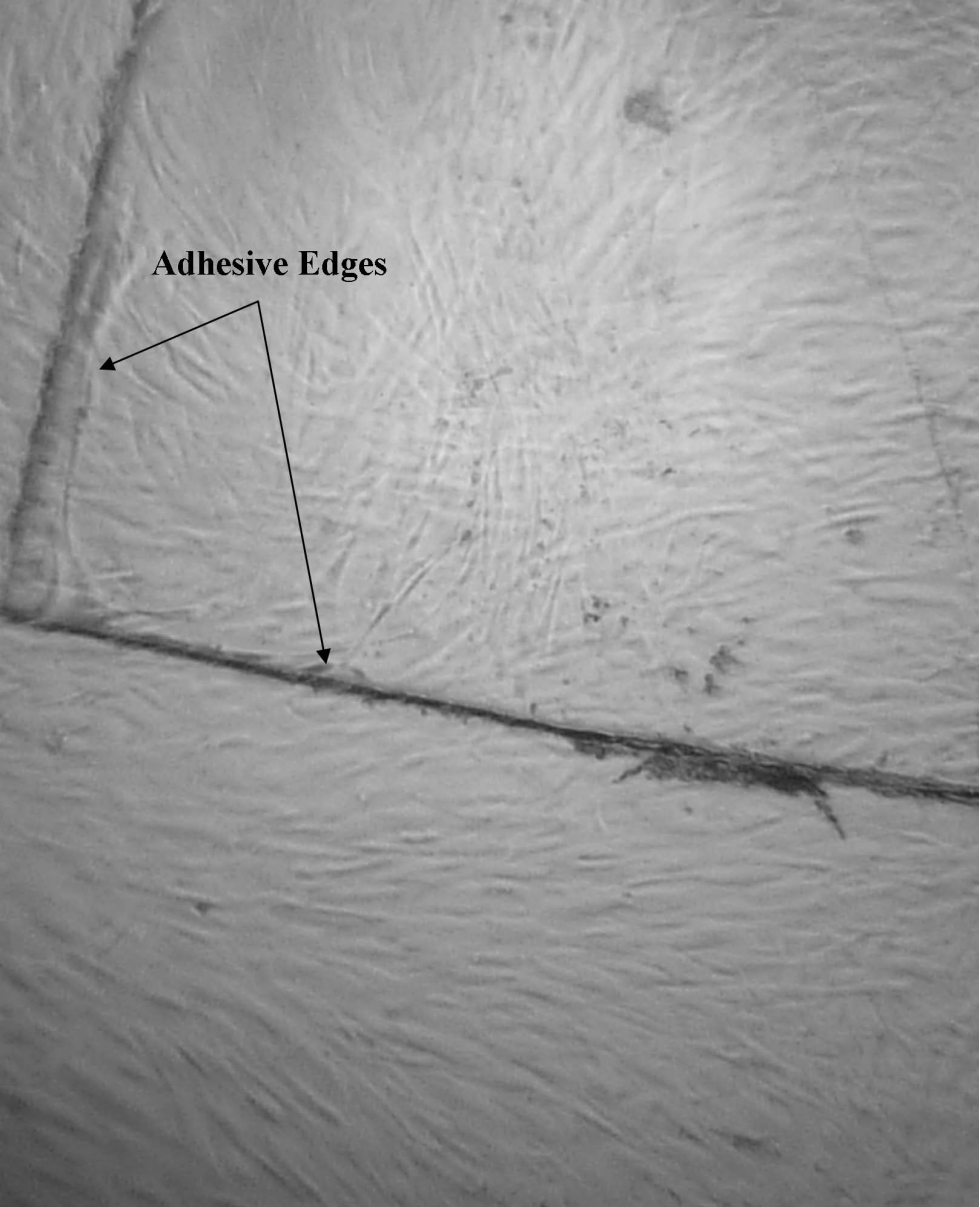
**Human fibroblasts grew confluent on the rose adhesive and on the culture well**. No morphological change could be detected in these cells under the microscope.

After 48 h of incubation, fibroblasts grew confluent on the rose adhesive and on the adjacent culture well (Figure 5). No morphologic changes were observed under the microscope in the cells attached to the adhesive when compared to fibroblasts in the control wells. The adhesive did not produce any significant toxic effect on cells.

## Discussion

Photochemical tissue bonding is an alternative technology to suturing that avoids tissue thermal damage. Redmond *et al. *[7] performed successfully femoral artery anastomosis in rats dispensing RB (0.1% w/v in phosphate buffer solution) between the vessel walls and irradiating with a green laser (Fluence 70-134 J/cm^2^, Irradiance ~0.5 W/cm^2^). The bonded tissue could withstand a pressure of 146 ± 20 kPa. At 8 weeks post-repair the patency rate was 80% and there was no evidence of aneurysm formation or bleeding. The histology of the operated aorta did not show any sign of thermal injury acutely and after 8 weeks. This outcome was in agreement with the direct measure of tissue temperature during PTB of porcine skin grafts [8]. The grafts were treated with a RB solution (0.1% w/v), approximated dermis-to-dermis and irradiated with an argon laser at 514 nm. Graft adhesion was successful and the skin surface temperature increased from 23.0 to 31.2°C after 60 s of irradiation at the irradiance level of 0.56 W/cm^2 ^[8]. The basic principles of PTB have been applied in the present study to chitosan films. In our experiments, the rose bengal was incorporated in the films, which were irradiated by a green laser (I = 0.9 W/cm^2 ^, F = 110 J/cm^2^). These films bonded firmly to the intestine (~15 kPa) while their temperature increased from 26 to 32°C in 30 s. From the visible spectra of Figure 3, it appears that no significant aggregation of RB occurred in the adhesive films; consequently, the quantum yield of RB (as a photochemical sensitizer) was not considerably affected during the adhesive fabrication. The bonding mechanism of the rose adhesive is not clear yet, although the RB ability of producing singlet oxygen, upon light irradiation, may play a role in crosslinking collagen and chitosan via amino groups [21]. It should be recalled that during the temperature measures the rose adhesive was irradiated with ~5.7 W/cm^2^, which was 6 times higher than the irradiance (~0.9 W/cm^2^) used to repair the intestine. Any thermal effect would have been amplified by this high irradiance [8]. In other studies [18,22], the chitosan adhesive, containing IG (0.02% w/v), was laser-activated exothermally at 60-65°C to bond to tissue (~13 kPa). This bonding strength was achieved using a beam fluence and irradiance of ~49 J/cm^2 ^and ~15 W/cm^2^, respectively. The comparison of the chitosan adhesives incorporating IG or RB appears problematic: the laser parameters applied to the two adhesives are significantly different because their adhesion mechanism is different. It appears from a qualitative assessment that their bonding strengths are similar. A dedicated study is necessary to evaluate more rigorously this matter.

The rose adhesive had no significant toxic effect on human fibroblasts, which were successfully plated on it as shown in Figure 5. The fibroblasts attached to the adhesive were morphologically similar to the cells attached on the culture well. The rose adhesive allowed cell attachment and growth in agreement with previous reports where chitosan films proved to be biocompatible and not cytotoxic [18,23]. Our study showed that the RB concentration in the adhesive was safe and the dye did not leak out from the adhesive in the cell medium at a toxic concentration. Mousavi *et al. *[24] reported that 100 μM of RB dissolved in cell medium did not induced significant reduction in the viability of HFSF-P13 non-malignant cells. RB reduced the viable cell number to 90% and 80% at a concentration of 200 μM and 300 μM respectively, after 48 hours incubation. In our study, the initial concentration of RB in the chitosan solution was 0.014% w/v (140 μM). The water content in the rose adhesive is similar to previously reported values for chitosan adhesives (~10 wt%) [18], thus the [RB] in the films can be estimated to be ~1.4 mM (10× higher the concentration in solution). The rose adhesive should not have significant toxic effects in the body as tissue is more resilient then cells to photochemical damage [25]. PTB had indeed negligible adverse effects when a RB solution of 1 mM was used to repair tissue [7,9,25]. The concentration of RB and adhesive thickness can be optimized to allow more radiation at the tissue interface and possibly enhance the bonding strength. A thinner film would, for example, increase the laser irradiance and fluence at the adhesive- tissue interface. However, care should be taken in reducing the film thickness in order to prevent excessive heating of tissue during laser irradiation.

The rose adhesive has a promising use in repairing soft tissue inside the body, such as peripheral nerves [9,15]. It has also applications in tissue engineering. It can be integrated, for example, in a bandage with extracellular matrices to repair tissue and enhance wound healing without the aid of sutures. In our recent study [22], a bandage was fabricated with small intestine submucosa and chitosan films, incorporating IG. The bandage adhered tightly to tissue upon laser irradiation but the adhesive temperature increased to ~ 60°C, exposing tissue to possible injury. The rose adhesive will allow the fabrication of a similar bandage that is laser-activated without significant temperature rise and tissue thermal damage. Chitosan is often used to fabricate scaffolds for clinical procedures; nevertheless a non invasive method to anchor them to the target tissue is sought. The PTB technique described in this study may assist the bonding of chitosan scaffolds to tissue without the use of sutures or staples.

## Conclusions

The rose adhesive proved to bond firmly to tissue upon laser exposure with minimal temperature increase and heat production. For this reason, the rose adhesive represents a major advancement when compared to exothermal solders and adhesives. Future studies are required to validate the efficacy of the rose adhesive for tissue repair in animal models.

## Competing interests

The authors declare that they have no competing interests.

## Authors' contributions

LA conceived and designed the study. LA fabricated the rose adhesive and performed the tissue repair studies with the assistance of MD, JH, AG and BM. The cell study was done by PS and BM. All Authors have contributed and approved the final manuscript.
